# The PRMT5/WDR77 complex restricts hepatitis E virus replication

**DOI:** 10.1371/journal.ppat.1011434

**Published:** 2023-06-05

**Authors:** Xiaohui Ju, Yanying Yu, Wenlin Ren, Lin Dong, Xianbin Meng, Haiteng Deng, Yuchen Nan, Qiang Ding

**Affiliations:** 1 School of Medicine, Tsinghua University, Beijing, China; 2 School of Life Sciences, Tsinghua University, Beijing, China; 3 Department of Preventive Veterinary Medicine, College of Veterinary Medicine, Northwest A&F University, Yangling, China; National Institute of Allergy and Infectious Diseases, UNITED STATES

## Abstract

Hepatitis E virus (HEV) is one of the main pathogenic agents of acute hepatitis in the world. The mechanism of HEV replication, especially host factors governing HEV replication is still not clear. Here, using HEV ORF1 *trans*-complementation cell culture system and HEV replicon system, combining with stable isotope labelling with amino acids in cell culture (SILAC) and mass spectrometry (MS), we aimed to identify the host factors regulating HEV replication. We identified a diversity of host factors associated with HEV ORF1 protein, which were putatively responsible for viral genomic RNA replication, in these two cell culture models. Of note, the protein arginine methyltransferase 5 (PRMT5)/WDR77 complex was identified in both cell culture models as the top hit. Furthermore, we demonstrated that PRMT5 and WDR77 can specifically inhibit HEV replication, but not other viruses such as HCV or SARS-CoV-2, and this inhibition is conserved among different HEV strains and genotypes. Mechanistically, PRMT5/WDR77 can catalyse methylation of ORF1 on its R458, impairing its replicase activity, and virus bearing R458K mutation in ORF1 relieves the restriction of PRMT5/WDR77 accordingly. Taken together, our study promotes more comprehensive understanding of viral infections but also provides therapeutic targets for intervention.

## Author summary

HEV is an underestimated pathogen, causing about 20 million infections worldwide every year and leading to approximately 70,000 deaths. There are no direct-acting antivirals for HEV treatment. Although significant progress has been made about HEV replication, but host factors regulating HEV replication have not been fully characterized. Here, using ORF1 *trans*-complementation system and HEV replicon harbouring HA-Flag tag in ORF1, we systematically analyse host factors in HEV replication complex in viral replication condition. Specifically, protein arginine methyltransferase 5 (PRMT5)/WDR77 complex was found to be in HEV replication complex and play an inhibitory role in HEV infection among different HEV strain, but not in HCV and SARS-CoV-2 infection. In details, PRMT5/WDR77 complex is able to methylate HEV ORF1 at 458^th^ arginine, which is responsible for HEV replication, to repress HEV replication. Consistently, HEV with R458K substitution in ORF1 escape the restriction by PRMT5/WDR77, with enhanced replication capability. This work not only provides novel insights into HEV replication and viral-host interaction, but also informs the antiviral strategy against HEV infection.

## Introduction

It is estimated that more than 20 million people are infected with HEV worldwide annually, in which 3.4 million are symptomatic, causing 70 thousand deaths and 3 thousand stillbirths [1,2]. Usually, HEV infection only causes acute infection in healthy individuals, which is self-limiting. However, in immunocompromised people such as organ-transplant recipients [3,4] and human immunodeficiency virus (HIV) [5,6] infected individuals, HEV can result in chronic infection, and these patients are at a higher risk of developing rapid progression to cirrhosis. Unfortunately, there is no direct-acting antivirals to treat HEV infection up to now. Ribavirin and pegylated interferon are only available drugs to treat HEV infection [7]. However, emerging of ribavirin-resistant HEV strains challenges its efficacy [8]. It is urgent to obtain comprehensive understanding about HEV life cycle, especially host factors regulating HEV replication, which can be used as drug targets to develop antivirals against HEV infection potentially [9].

HEV belongs to *Hepeviridae* family and the *Paslahepevirus* genus. Members of the species *Paslahepevirus balayani* have been assigned to 8 genotypes, HEV-1 to HEV-8 [10,11]. Among these genotypes, HEV-1, HEV-2, HEV-3, HEV-4, and HEV-7 are most commonly associated with HEV infection in humans [11,12]. The genome of HEV is a positive-sense, single-stranded RNA of 7.2 kb in length, which contains 5’ cap and 3’ poly(A) tail. There are three proteins encoded by HEV genome, ORF1 (replicase) [13], ORF2 (capsid) [14,15] and ORF3 (ion channel) [16]. The replication of HEV genome needs ORF1 non-structural protein, template RNA and host factors participating in this process [9]. Previously, whether ORF1 needs to be processed to achieve its function is controversial [7,17]. Therefore, most studies identifying host factors interacted with HEV replicase were performed by expressing ORF1 different domains individually (Met, PCP, Hel, RdRp etc.) but not full-length ORF1 [18,19]. However, recent studies with functional ORF1 *trans*-complementation system [20,21] and recombinant HEV harbouring tags in the ORF1 [22], revealed that only full-length ORF1 are detected by both ORF1 and HA antibody in these two systems, suggesting that unprocessed ORF1 is functional to replicate HEV genome. Another study had studied tagged full-length ORF1 in heterologous, replicon and infectious systems, and it is reported that the full-length ORF1 protein and less abundant products of lower molecular weight were detected, suggesting processing of ORF1 may occur [23].

Protein arginine methyltransferase 5 (PRMT5) and WDR77 (or MEP50) can form a complex to catalyse protein arginine methylation [24–26]. PRMT5 and WDR77 have been reported to regulate alternative splicing by catalysing methylation of splicing factors, for example, SRSF1 [27]. Besides, PRMT5 also methylates histones to regulate gene expression [28]. Previous studies have demonstrated that PRMT5/WDR77 complex plays various roles in different virus infection, including human respiratory syncytial virus (HRSV) [29], human immunodeficiency virus (HIV) [30] and hepatitis B virus (HBV) [31].

In this study, we systematically identified host factors associated with ORF1 in ORF1 *trans*-complementation and HA-Flag tagged ORF1 HEV replicon cell culture models. The host proteins associated with ORF1 identified in these two cell culture systems were highly consistent. We found that KIF11, ANKFY1, STK38, HSD17B10, KCTD5, PRMT5 and WDR77 were components of HEV replication complex. Specifically, PRMT5/WDR77 complex is able to catalyse methylation on R458 of ORF1 to inhibit HEV replication. Consistently, R458K mutation in ORF1 can compromise PRMT5/WDR77 restriction, and knockdown of PRMT5 reduced methylation of R458 of ORF1. Our findings not only facilitate our understanding of HEV replication but also provide a novel antiviral target of HEV infection.

## Results

### Identification of cellular proteins interaction with HEV ORF1 protein by proteomics approaches

HEV ORF1 protein is responsible for HEV genome replication [32,33], and cellular factors interacting with ORF1 could potentially regulate HEV replication. To discover the cellular proteins governing HEV replication, we employed two HEV cell culture models recapitulating viral replication, combined with stable isotope labeling by amino acids in cell culture (SILAC) [34,35] and affinity purification coupled with mass spectrometry (AP-MS) proteomic approaches, for identification of the host proteins associated with ORF1 protein.

**Fig 1:**
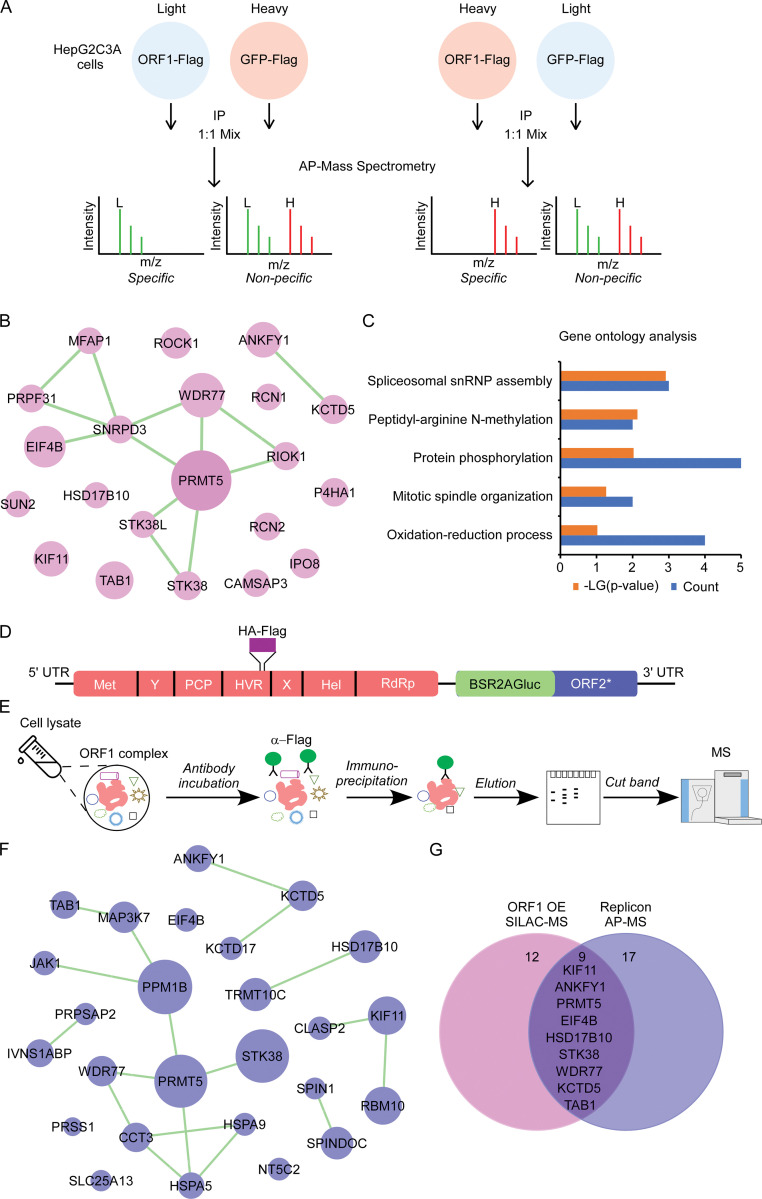
Identifications of the interaction partners of HEV replication complex in ORF1 *trans*-complementation and replicon systems by proteomics approaches. (A) Schematic representation of how SILAC and mass spectrometry identify host proteins interacting with ORF1 specifically. Cells expressing GFP-Flag or ORF1-Flag are cultured in “Heavy” or “Light” culture medium. Then, cells are lysed, immunoprecipitated with Flag antibody and analyzed by mass spectrometry. Proteins specifically appeared in ORF1 co-immunoprecipitated sample at high abundance were components of replication complex. (B) Interaction network of host factors associated with ORF1 (ORF1/GFP fold change>3 and score>5) was analyzed by STRING [60], and was drawn with Cytoscape. (C) Gene ontology analysis of host factors interacting with ORF1 was performed with the database for annotation, visualization and integrated Discovery (DAVID) [61]. (D) Schematic representation of Kernow C1/p6 ORF1HA-Flag BSR2AGluc replicon in which ORF1 is tagged with HA-Flag. (E) Workflow of purification and characterization of ORF1 replication complex with HEK293T Kernow C1/p6 ORF1HA-Flag BSR2AGluc replicon cells. (F) Interaction network of host proteins in ORF1 replication complex was analyzed by STRING, and was drawn with Cytoscape. (G) Comparison of proteins interacting with ORF1 in ORF1 *trans*-complementation and Kernow C1/p6 ORF1HA-Flag BSR2AGluc replicon systems.

Our previous studies have demonstrated that ORF1 could function *in trans* to replicate viral genome [20,21]. Thus, we transduced HEV-permissive hepatoma cell HepG2C3A expressing GFP-Flag or ORF1-Flag by lentivirus (**S1A Fig**) and the ectopic ORF1 could replicate HEV genome (**S1B Fig**), consistent with our previous results [20,21]. To identify cellular proteins specifically associated with functional ORF1, we performed quantitative proteomics analysis using SILAC, in which cells are labeled through the incorporation of stable “light” or “heavy” versions of essential amino acids (lysine and arginine) [34,35]. After 15 days culturing in the SILAC medium to attain complete labeling, we proceeded to identify the cellular proteins using affinity purification followed by mass spectrometry (MS) analysis **(Fig 1A**). Flag tagged ORF1 or GFP were immunoprecipitated by anti-Flag magnetic beads in parallel and the purified samples were subsequently subjected to SDS-PAGE analysis. As expected, Flag tagged ORF1 and GFP were highly enriched; notably, specific bands in ORF1 co-immunoprecipitation sample but not GFP sample were visible (**S1C Fig**), suggesting that host factors specifically associated with ORF1 were present in the samples. All the samples were subsequently subjected to Orbitrap Fusion mass spectrometer analysis after trypsin digestion. MS identified a diverse number of host proteins specifically associated with ORF1, including PRMT5, WDR77, ROCK1 and SNRPD3 etc. (**Fig 1B** and **S1 Table**). Gene ontology analysis showed that spliceosome components, protein methylation and phosphorylation genes were enriched, implying their roles in HEV replication (**Fig 1C**).

In parallel, we also analysed the components of replication complex in viral replication condition for identification of the host proteins which presumably governing viral replication. To this end, we constructed HEV replicon harbouring HA-Flag tags (Kernow C1/p6 ORF1HA-Flag BSR2AGluc replicon) within the hypervariable region (HVR) of ORF1 protein according to previous study [22] (**Fig 1D**). Then, the *in vitro* transcribed Kernow C1/p6 ORF1HA-Flag BSR2AGluc replicon RNAs were transfected into HEK293T cells and blasticidin (BSD) was subsequently supplemented in the culture medium to select HEK293T with active HEV replication. The insertion of HA-Flag tag has negligible impact on viral replication, as evidenced by comparable luciferase activities between WT and Kernow C1/p6 ORF1HA-Flag BSR2AGluc replicon (**S1D Fig**). In addition, ORF1 expression was analysed by immunoblotting assay, and only one high molecular weight protein (above 180 kDa) was detected by Flag antibody corresponding to the unprocessed form of ORF1 (**S1E Fig**), consistent with previous observations [22]. To capture viral replication complex, we lysed the replicon cells and cells expressing Flag tagged SARS-CoV-2 nucleocapsid protein (N-Flag) as the control and ORF1-Flag or N-Flag were subsequently immunoprecipitated by Flag antibody. The samples were then analysed by SDS-PAGE and the proteins specifically present in ORF1-Flag sample were excised from the gel and subjected to Orbitrap Fusion mass spectrometer analysis after trypsin digestion **(Figs 1E** and **S1F**). MS analysis revealed more than 26 host proteins in HEV replication complex, including PRMT5, WDR77, PPM1B, KCTD5 and STK38 etc (**Fig 1F** and **S2 Table**). Comparing host proteins associated with ORF1 in ORF1 *trans*-complementation (**Fig 1B**) and replicon systems (**Fig 1F**), nine host proteins were identified using both approaches, including PRMT5, WDR77, KIF11, ANKFY1, EIF4B, HSD17B10, STK38, KCTD5 and TAB1 (**Fig 1G**). It is conceivable that these proteins were associated with ORF1 and regulated HEV replication.

### Validation of PRMT5/WDR77 complex interacting with HEV ORF1

**Fig 2:**
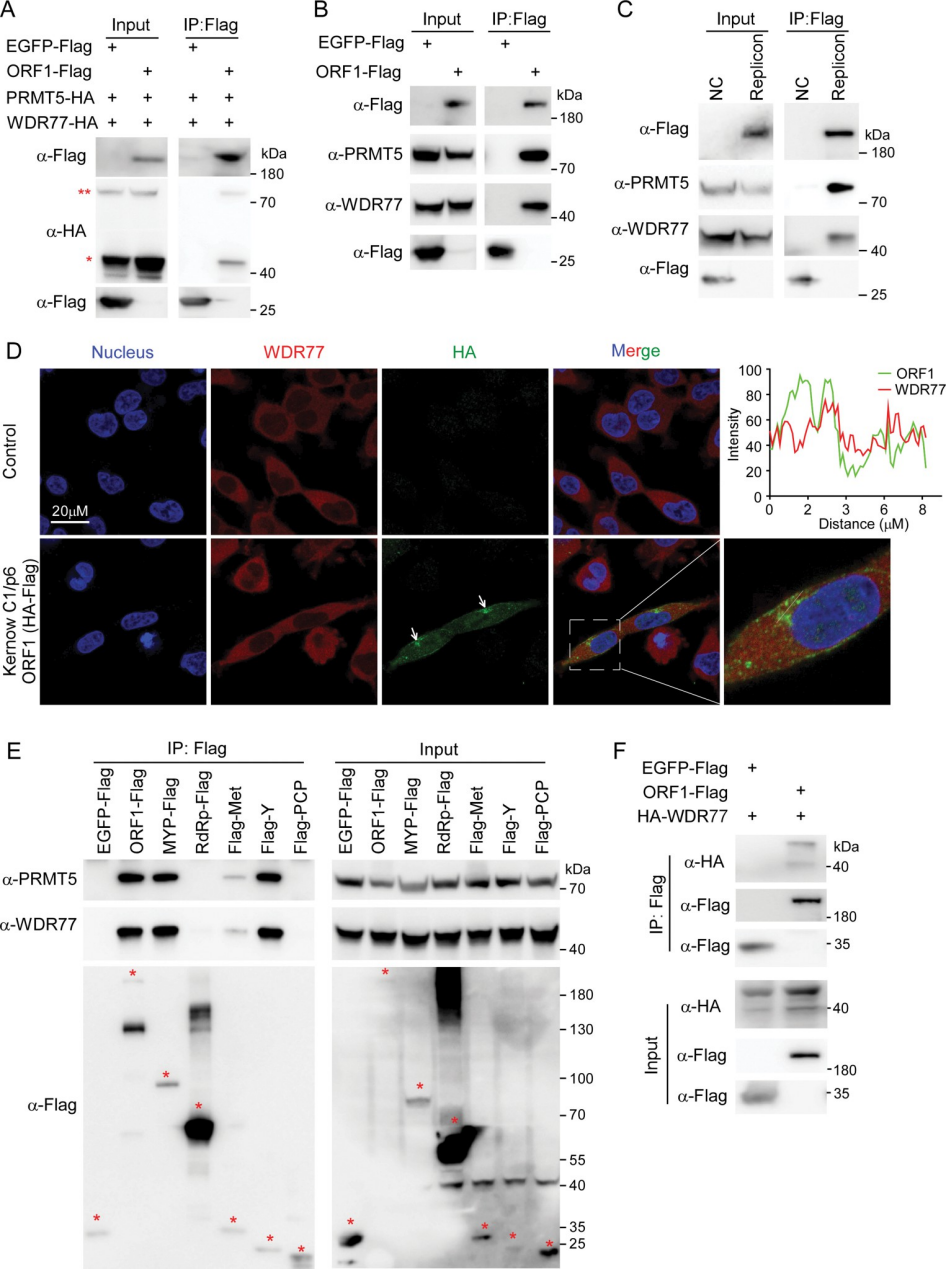
PRMT5/WDR77 complex interacts with ORF1. (A) Co-IP assay of ORF1-Flag, WDR77-HA and PRMT5-HA. HEK293T cells were co-transfected with ORF1-Flag or EGFP-Flag and WDR77-HA, PRMT5-HA constructs. Cell lysates were incubated with Flag antibody. Immunoprecipitated samples were blotted with Flag and HA antibody. One star indicates WDR77 and two stars indicate PRMT5. (B) Co-IP assay of ORF1-Flag and endogenous PRMT5, WDR77. HEK293T cells was transfected with ORF1-Flag or EGFP-Flag plasmid, and cell lysates were incubated with Flag antibody. Immunoprecipitated sample were blotted with Flag, PRMT5 and WDR77 antibodies. (C) Western blot analysis of ORF1 replication complex purified from HEK293T Kernow C1/p6 ORF1HA-Flag BSR2AGluc replicon cells. Replication complex was immunoprecipitated with Flag antibody and blotted with Flag, PRMT5 and WDR77 antibodies. HEK293T cells expressing EGFP-Flag were included as the negative control. (D) Immunofluorescence of ORF1 and WDR77 in HEK293T Kernow C1/p6 ORF1HA-Flag BSR2AGluc replicon cells. Cells were stained with HA (green) and WDR77 (red) antibodies prior to analysis by confocal microscopy. Perinuclear puncta-like structures are shown (white arrowheads). The cell nuclei were stained with DAPI (blue). Line profiles corresponding to the white lines show colocalization. (E-F) Co-IP assay of ORF1 full-length, truncations and PRMT5, WDR77 or HA-WDR77. Flag tagged ORF1 full-length, its truncations or EGFP-Flag was expressed in HEK293T cells and immunoprecipitated by Flag antibody. Immunoprecipitated sample were blotted with Flag, PRMT5, WDR77 and HA antibodies. All data are representative of three independent experiments.

Among the host factors associated with ORF1 protein, protein arginine methyltransferase 5 (PRMT5) and WDR77 were top candidates in both ORF1 *trans*-complementation and replicon systems (**Fig 1B, 1F** and **1G**), and it has been reported that PRMT5 is complexed with the WD repeat protein WDR77 as a tetramer of heterodimers to catalyse arginine methylation regulating protein function [36] (**S2A Fig**). WDR77 is required for the recruitment of protein substrates to the catalytic domain of PRMT5 [36,37]. We next sought to dissect the function of PRMT5/WDR77 complex in HEV life cycle. First, we performed co-immunoprecipitation (Co-IP) assays to test the interaction of PRMT5/WDR77 with HEV ORF1 protein. We ectopically expressed HA tagged WDR77 and PRMT5, together with Flag tagged ORF1 or EGFP in HEK293T cells. After 48h, cell lysates were collected and immunoprecipitated with Flag antibody for immunoblotting analysis. Our results showed that PRMT5-HA and WDR77-HA can be co-immunoprecipitated by ORF1-Flag but not EGFP-Flag (**Fig 2A**), suggesting PRMT5 and WDR77 could specifically interact with HEV ORF1. To confirm the interaction between endogenous PRMT5/WDR77 and HEV ORF1, we overexpressed ORF1-Flag or EGFP-Flag and performed Co-IP assay in HEK293T cells. As expected, HEV ORF1 could specifically interact with endogenous PRMT5/WDR77 (**Fig 2B**). To establish whether ORF1 could associate with PRMT5/WDR77 in the context of viral replication, we collected the cell lysates of Kernow C1/p6 ORF1HA-Flag BSR2AGluc replicon cells and performed immunoprecipitation using Flag antibody. Of note, the endogenous PRMT5/WDR77 could associate with viral ORF1 in the context of viral replication (**Fig 2C**). In addition, we performed immunofluorescence analysis of HEV ORF1 and endogenous WDR77 in the HEK293T cells harbouring Kernow C1/p6 ORF1HA-Flag BSR2AGluc replicon RNA (**Figs 1D** and **S1D**). Consistent with previous observation, ORF1 predominantly localized in the cytosol as perinuclear foci (**Fig 2D,** white arrowheads), which was considered as HEV viral factory [23]. Interestingly, the endogenous WDR77 staining overlapped with ORF1 perinuclear foci, suggesting that WDR77 could co-localized with HEV ORF1 in the viral factory.

To further dissect which domain of ORF1 could interact with PRMT5/WDR77 complex, we transfected MYP (Met, Y and PCP domain)-Flag, Flag-Met, Flag-Y, Flag-PCP, RdRp-Flag, ORF1-Flag or EGFP-Flag into HEK293T cells respectively, and then performed Co-IP assay. We found that MYP-Flag, Flag-Y and ORF1-Flag could specifically interact with PRMT5/WDR77 complex (**Fig 2E**), suggesting that Y domain of ORF1 is responsible for the interaction of ORF1 with PRMT5/WDR77 complex. WDR77 recruits the substrate to PRMT5 for methylation [24]. To test whether ORF1 could interact with WDR77, we performed Co-IP experiment by co-transfection of ORF1-Flag with HA-WDR77 into HEK293T cells, and EGFP-Flag was included as the control. Our results showed that WDR77 specifically interacted with ORF1 (**Fig 2F**). In addition, HEV infection did not alter the mRNA abundance of WDR77 and PRMT5 (**S2B Fig**), also has negligible effect on the amount of WDR77 protein and its localization (**S2C and S2D Fig**).

Taken together, these results collectively demonstrated that PRMT5/WDR77 complex was associated with HEV ORF1, indicating that PRMT5/WDR77 complex could regulate viral replication.

### The PRMT5/WDR77 complex restricts HEV infection

**Fig 3:**
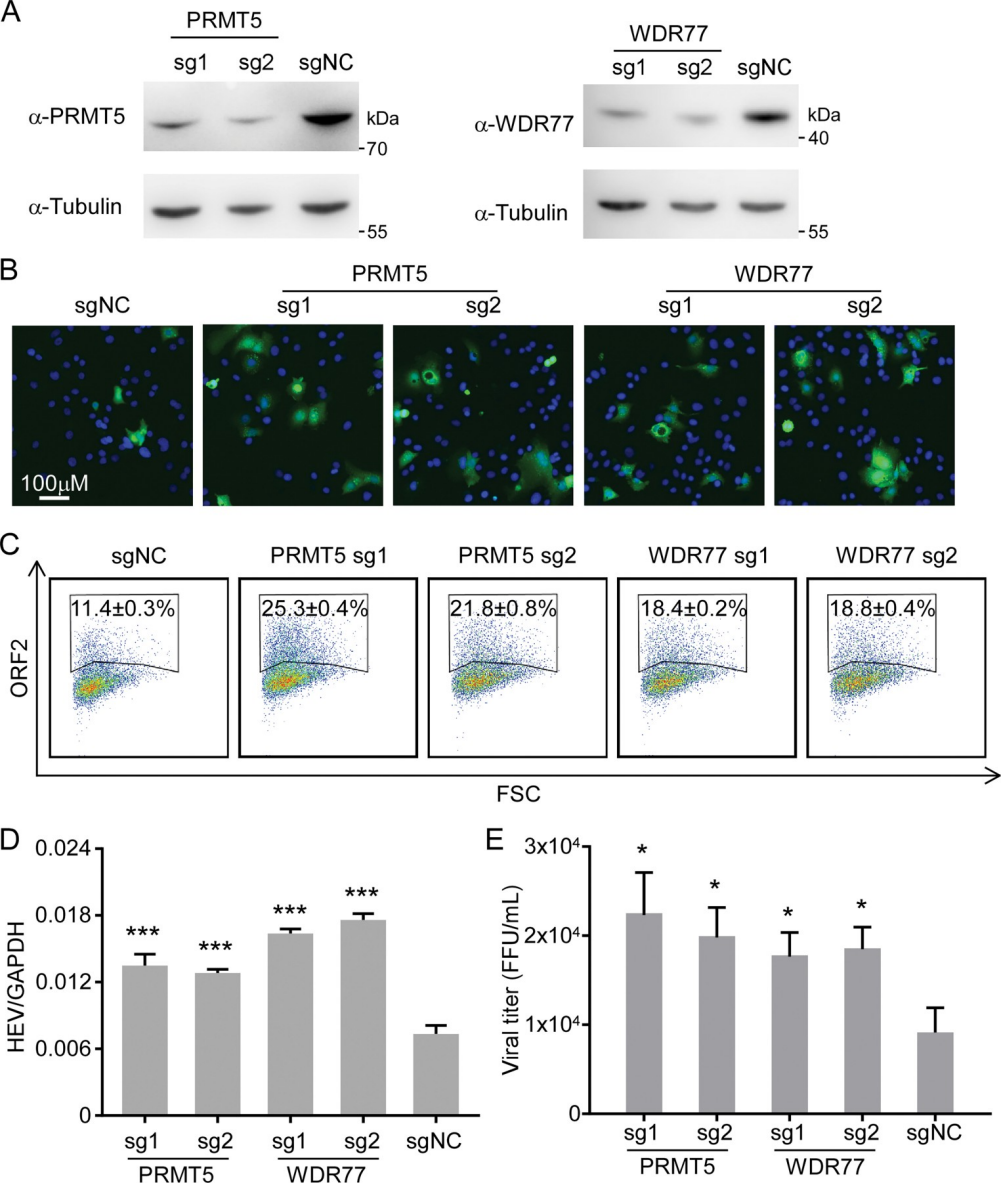
PRMT5/WDR77 complex inhibits HEV infection. (A) Western blot analysis of lysates from HepG2C3A cells transduced with PRMT5, WDR77 or non-targeting control plentiCRISPRv2 sgRNA lentivirus. Tubulin was used as the loading control. (B) HepG2C3A cells were infected with HEV Kernow C1/p6 virus. Immunofluorescence of ORF2 was performed at day 3 post infection with ORF2 monoclonal antibody (2G8) [20]. (C) Flow cytometry analysis of HEV infection by ORF2 staining using 2G8 antibody was performed at day 3 post infection. (D) RT-qPCR analysis of HEV genomic RNAs at day 3 post infection. (E) Intracellular viral titer of Kernow C1/p6 was determined at day 7 post transfection in S10-3 cells. Values are means plus standard deviations (SD) (error bars) (n = 3). *, P < 0.05; ***, P < 0.001 by one-way ANOVA. All data are representative of three independent experiments.

To investigate the role of PRMT5/WDR77 complex in HEV replication, we utilized CRISPR/Cas9 system [38] to knockdown PRMT5 or WDR77 in HepG2C3A cells. We utilized two independent sgRNAs for each gene to exclude the off-target effect. Immunoblotting assay was performed to confirm the efficiency of PRMT5 and WDR77 knockdown (**Fig 3A**). Next, we infected the WT or knockdown HepG2C3A cells with recombinant HEV Kernow C1/p6 virus, and the virus infection was assessed by immunostaining, flow cytometry analysis, RT-qPCR and titration assays after three days of infection. Immunostaining of viral ORF2 antigen by 2G8 antibody [20] suggested that Kernow C1/p6 infection was enhanced upon PRMT5 or WDR77 knockdown (**Fig 3B**), which was also supported by the flow cytometry data (**Fig 3C**) as evidenced by increased HEV infection (ORF2 positive cells) upon PRMT5 and WDR77 knockdown. Moreover, viral RNA levels determined by RT-qPCR assay were also increased in the PRMT5 or WDR77 knockdown cells in comparison with WT cells (**Fig 3D**). In addition, PRMT5 or WDR77 knockdown led to the increased virus production compared with WT cells (**Fig 3E**). These results collectively suggest that PRMT5/WDR77 complex could restrict HEV infection.

**Fig 4:**
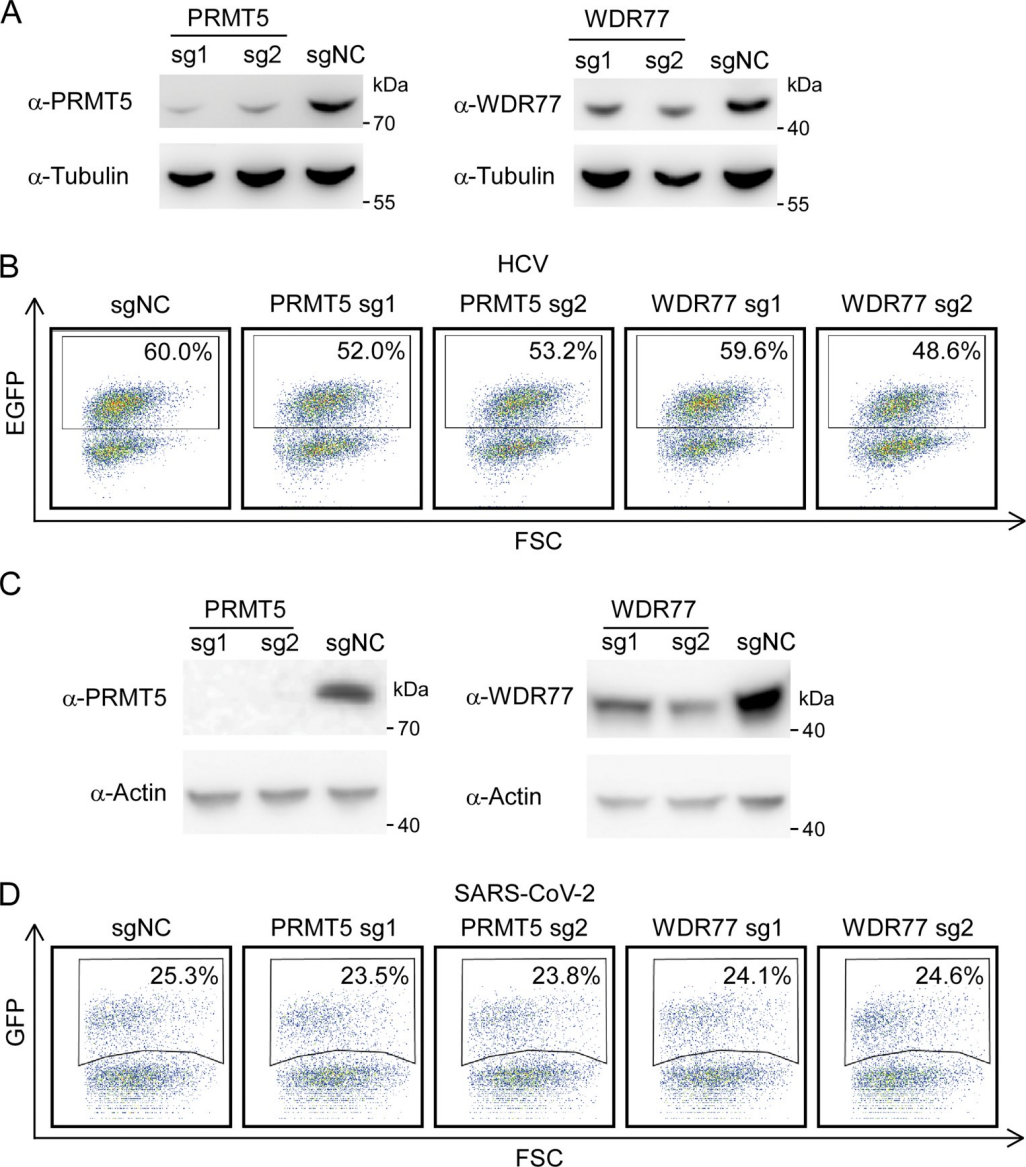
PRMT5/WDR77 complex could not restrict HCV and SARS-CoV-2 infection. (A) Western blot analysis of lysates from S10-3 cells transduced with PRMT5, WDR77 or non-targeting control sgRNA lentivirus. Tubulin was used as the loading control. (B) Flow cytometry analysis of EGFP expression at day 4 post HCV-NS5A-EGFP infection at MOI of 0.1. (C) Western blot analysis of lysates from Caco-2-N cells transduced with PRMT5, WDR77 or non-targeting control sgRNA lentivirus. Actin was used as the loading control. (D) Flow cytometry analysis of GFP expression at 24 hours post SARS-CoV-2 GFP/ΔN trVLP infection at MOI of 0.05. Data in all panels were representative of three independent experiments.

To investigate whether PRMT5/WDR77 complex could restrict other RNA virus infection, we tested its restriction on hepatitis C virus (HCV) and severe acute respiratory syndrome coronavirus 2 (SARS-CoV-2). S10-3, the subclone of Huh7 cells permissive for HCV and HEV infection [39], were knockdown with PRMT5 or WDR77 by CRISPR/Cas9 mediated gene editing (**Fig 4A**). The S10-3 WT or knockdown cells were subsequently infected with HCV-EGFP reporter virus (MOI = 0.1) [40] or HEV Kernow C1/p6 virus (MOI = 0.1), EGFP or HEV ORF2 expression was analyzed by flow cytometry at day four post infection. Our results showed that HEV infection of S10-3 cells was increased about two folds upon PRMT5 knockdown (**S3 Fig**), in line with the results of HepG2C3A cells (**Fig 3C**); in contrast, HCV infection decreased slightly after PRMT5 or WDR77 depletion (**Fig 4B**). Using transcription and replication-competent SARS-CoV-2 virus-like-particles (trVLP) cell culture model [41–43], we assessed the role of PRMT5 and WDR77 in SARS-CoV-2 infection. The Caco-2 cells expressing SARS-CoV-2 nucleocapsid (Caco-2-N) were depleted with PRMT5 or WDR77 (**Fig 4C**), and the WT or knockdown Caco-2-N cells were infected with SARS-CoV-2 trVLP at MOI of 0.05. After 24h, the trVLP infection was evaluated by quantification of GFP expression using flow cytometry. Our data revealed that trVLP infection of PRMT5 or WDR77 knockdown cells was comparable with that of WT cells (**Fig 4D**).

Collectively, these data demonstrate that PRMT5/WDR77 complex functions as a restriction factor to specifically inhibit HEV infection.

### The PRMT5/WDR77 complex could restrict HEV genomic RNA replication

**Fig 5:**
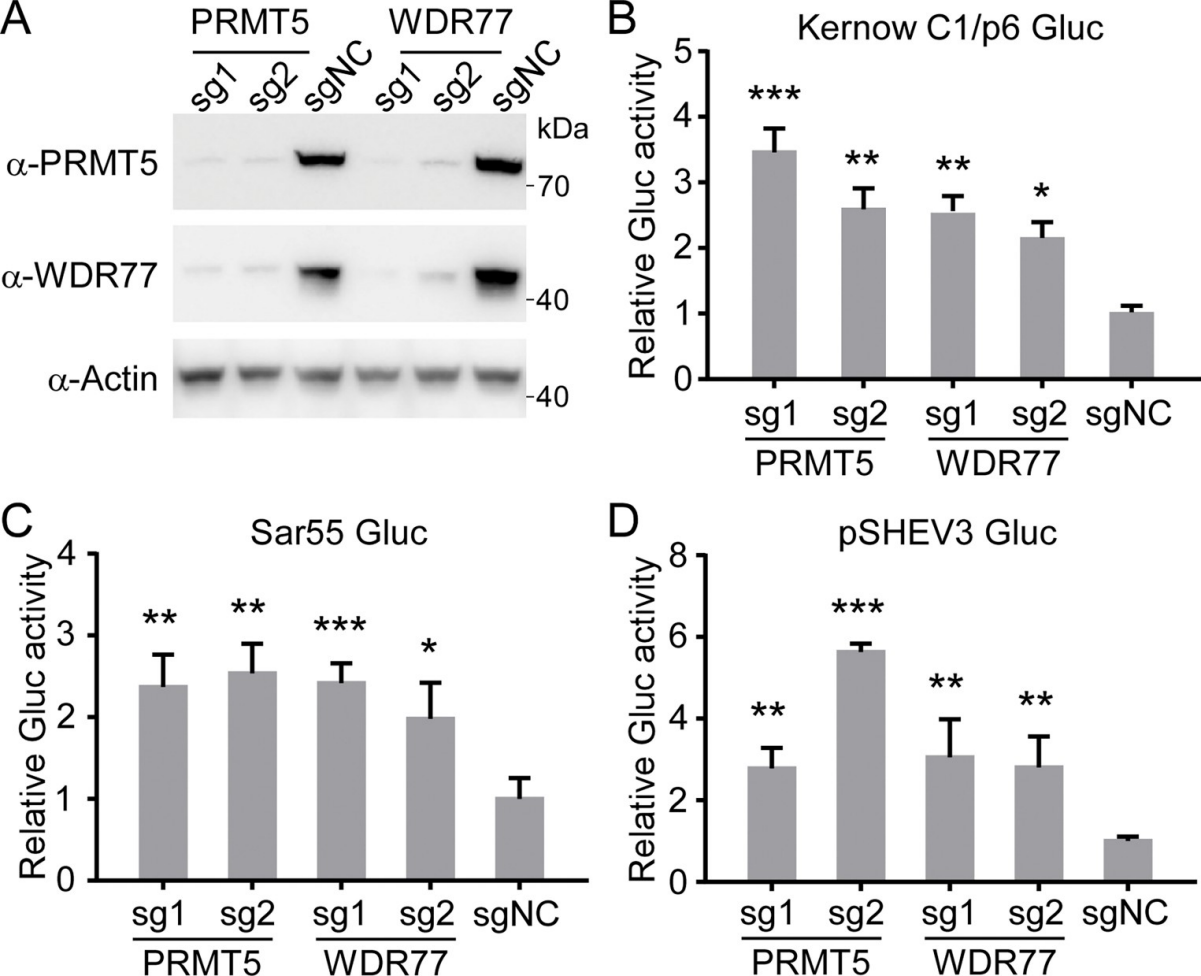
PRMT5/WDR77 complex restricts HEV genome replication. (A) Western blot analysis of lysates from S10-3 cells transduced with PRMT5, WDR77 or non-targeting control plentiCRISPRv2 sgRNA lentivirus. Actin was used as the loading control. (B-D) S10-3 cells were transfected with HEV replicon RNAs of Kernow C1/p6 Gluc replicon (B), Sar55 Gluc replicon (C) and pSHEV3 Gluc replicon (D). Supernatants were collected and Gluc activity was measured at day 2 post transfection. Data are normalized with non-targeting control sgRNA. Values are means plus standard deviations (SD) (error bars) (n = 3). *, P < 0.05; **, P < 0.01; ***, P < 0.001 by one-way ANOVA. All data are representative of three independent experiments.

Given the PRMT5/WDR77 complex restricts HEV infection (**Fig 3**) and they were also associated with HEV ORF1 (**Fig 2**), which is responsible for HEV genomic RNA replication, we thus hypothesize that PRMT5/WDR77 complex restricts HEV infection by inhibition of HEV genomic RNA replication. To test this, we depleted PRMT5 or WDR77 by CRISPR/Cas9 in S10-3 cells, and monitor HEV replication by transfection of HEV replicon RNA harboring Gluc reporter gene. Immunoblotting results showed that PRMT5 or WDR77 was successfully knockdown; in addition, knockdown of PRMT5 could lead to the instability of WDR77 and *vice versa* (**Fig 5A**), highlighting the interdependence of these proteins for stability, which had also been observed in previous studies [44,45]. Then, Kernow C1/p6 Gluc replicon RNAs (GT3) were transfected into WT or PRMT5/WDR77 knockdown cells. Two days of transfection, cell culture medium was collected to assay luciferase activity, then intracellular RNA was extracted to quantify viral RNA abundance by RT-qPCR assay. Interestingly, luciferase activity from PRMT5 and WDR77 knockdown cells was significantly higher than WT cells (**Fig 5B**), which was in line with the viral RNA abundance (**S4A Fig**), indicating that PRMT5/WDR77 restricts HEV genomic RNA replication.

To exclude strain or genotype-specific effect, we also examined the replication of SAR55-Gluc (GT1) [46] and pSHEV3-Gluc (GT3) [47] upon PRMT5/WDR77 knockdown. Consistent with that of Kernow C1/p6 Gluc (GT3), luciferase activity and viral RNA levels were increased in the PRMT5 or WDR77 knockdown cells (**Figs 5C–5D and S4B–S4C**). Altogether, these results demonstrate that PRMT5/WDR77 complex could inhibit HEV genomic RNA replication.

### PRMT5/WDR77 complex catalyzes ORF1 R458 methylation to inhibit HEV replication in a methyltransferase activity dependent manner

**Fig 6:**
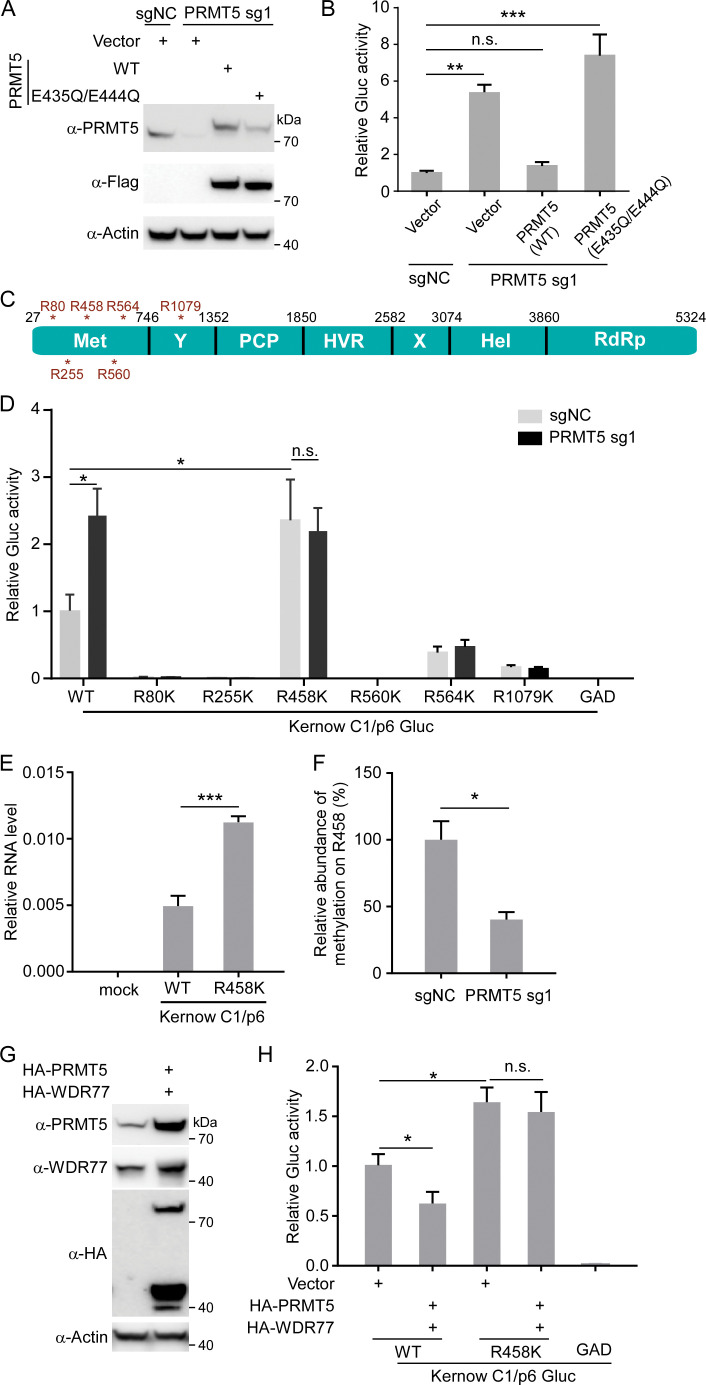
PRMT5/WDR77 complex catalyzes ORF1 R458 methylation to inhibit HEV replication in a methyltransferase activity dependent manner. (A) Western blot analysis of lysates from S10-3 PRMT5 knockdown cells transduced with cDNA constructs of PRMT5 WT or E435Q/E444Q mutant. (B) PRMT5 KO cells rescued with PRMT5 WT or E435Q/E444Q mutant were transfected with Kernow C1/p6 Gluc replicon RNAs. Supernatant was collected and Gluc activity was measured at day 2 post transfection. Gluc activity was normalized with non-targeting sgRNA expressing vector control. (C) Schematic representation of arginine methylation identified by mass spectrometry in ORF1. (D) Replicon RNAs of Kernow C1/p6 Gluc WT and mutants were transfected into WT S10-3 or PRMT5 knockdown cells, and luciferase activity was measured at day 2 post transfection. (E) S10-3 cells were transfected with Kernow C1/p6 WT or R458K RNAs and RT-qPCR was performed at day 7 post transfection. (F) Relative abundance of methylation on R458 in HEK293T WT and PRMT5 knockdown cells. The precursor abundance ratio at R458 was calculated as the abundance of methylated R458 divided by the total abundance of R458. The relative abundance of methylation in WT cells was set as 100%. (G) WB analysis of S10-3 cells overexpressing PRMT5 and WDR77. (H) S10-3 cells overexpressing PRMT5 and WDR77 were transfected with Kernow C1/p6 Gluc WT or R458K mutant replicon RNAs and Gluc activity was measured at day 2 post transfection. Values are means plus standard deviations (SD) (error bars) (n = 3). *, P < 0.05; **, P < 0.01; ***, P < 0.001; n.s., not significantly different by one-way ANOVA or Student’s t test. All data are representative of three independent experiments.

It has been reported that E435 and E444 of PRMT5 are critical for methyltransferase activity of the PRMT5/WDR77 complex, and E435Q/E444Q double mutations could impair PRMT5 catalytic activity [48]. We thus sought to test whether methyltransferase activity is required for PRMT5/WDR77 restriction on HEV replication. To this end, we overexpressed Flag tagged WT or E435Q/E444Q mutant of PRMT5 in PRMT5 KO S10-3 cells (**Fig 6A**). Then, Kernow C1/p6 Gluc replicon RNAs were transfected into these cells respectively, and Gluc activity was measured after 2 days. As expected, HEV replication was increased upon depletion of endogenous PRMT5 as evidenced by 5 folds increase of Gluc activity compared with WT cells (**Fig 6B**). However, overexpression of WT PRMT5 in the KO cells could dramatically inhibit HEV replication to the level comparable with that in the WT cells; in contrast, the E435Q/E444Q mutant did not exhibit restrictive activity for HEV replication (**Fig 6B**). These results demonstrated that methyltransferase activity of PRMT5 is required for its restriction on HEV replication.

PRMT5/WDR77 complex could catalyze the transfer of a methyl group from the co-factor S-adenosyl-l-methionine (AdoMet) to the arginine of a variety of substrates and regulate the substrates activity [24,25,48]. As PRMT5/WDR77 complex is associated with HEV ORF1 (**Fig 2A–2F**) and its methyltransferase activity is required for its restrictive activity against HEV infection (**Fig 6B**), we thus hypothesize that PRMT5/WDR77 complex catalyzes the methylations on arginine residue in ORF1 which impairs its replicase activity and subsequently restricts HEV replication. To test this hypothesis, we sought to identify the residues of ORF1 that are methylated by PRMT5/WDR77 complex. For this purpose, we performed mass spectrometry analysis of the methylation on arginine residues of the ORF1 protein. Among 124 arginine residues across the ORF1 protein, our analysis led to identification of six arginine residues which were methylated, such as R80, R255, R458, R560, R564, R1079 (**Figs 6C** and **S5**). To further identify the arginine residue(s) responsible for PRMT5/WDR77 complex restriction on HEV replication, we mutated the six arginine residues to lysine residues individually, which could not be methylated by PRMT5/WDR77 complex, and then monitored the replication of each mutant. The mutant replicon RNAs harboring each mutation were transfected into WT or PRMT5 knockdown S10-3 cells, and the Gluc activity was assayed after two days. As presented in **Fig 6D**, consistent with previous results that Kernow C1/p6 Gluc replicon RNA replicates more efficiently in PRMT5 knockdown cells than that of WT cells (**Fig 3C, 3D** and **3E**); among the six mutants, Kernow C1/p6 Gluc R458K replicon mutant exhibited enhanced replication as evidenced by increased Gluc activity about 2 folds in WT cells or PRMT5 knockdown cells than that of Kernow C1/p6 Gluc WT replicon in WT cells, but the R458K replicon mutant exhibited comparable replication with that of WT replicon in PRMT5 knockdown cells (**Fig 6D**) or WDR77 knockdown cells (**S6A Fig**), suggesting that the PRMT5 inhibits HEV replication by targeting R458. Other five replicon mutants were severely impaired in replication in WT cells or PRMT5 knockdown cells, suggesting that these mutations may impair viral replicative competence with unknown mechanisms (**Fig 6D**). In line with the replicon results, the full-length recombinant Kernow C1/p6 bearing R458K mutation possessed enhanced replication compared with WT virus, as evidenced by increased viral RNA following transfection of S10-3 cells (**Fig 6E**). In addition, the abundance of methylation for R458 was significantly reduced by knockdown of PRMT5 (**Fig 6F**). Furthermore, the ectopic expression of PRMT5/WDR77 could block Kernow C1/p6 Gluc WT replicon replication and viral titer (**Figs 6G**, **6H and S6B**); However, the R458K replicon mutant replication was dramatically elevated regardless of PRMT5/WDR77 overexpression compared with that of WT replicon RNA in WT cells (**Fig 6H**). We also analyzed the conservation of R458 across eight HEV genotype in the species of *Paslahepevirus balayani*, and sequence alignments of the R458 showed that this residue is conserved in seven genotypes, except in genotype two, which is serine (**S6C Fig**). Taken together, these results suggest that PRMT5/WDR77 complex methylated viral ORF1 at 458^th^ arginine residue, which impairs ORF1 replicase activity; substitution of 458^th^ arginine with lysine (R458K) relieved the restriction by PRMT5/WDR77 complex, thus the R458K mutant possesses enhanced replication.

## Discussion

HEV is one of the major causes of acute hepatitis, studies towards understanding of its pathogenesis, life cycle and virus-host interaction are urgently needed [9,17,49]. Cell receptor, which is a major genetic determinant of HEV infection and host range, has not been identified yet [50]. Numerous host factors, including translational initiation factors (such as EIF4A and EEF1A1) [18], microRNA-122 [51] and heterogeneous nuclear ribonucleoproteins [52] have been identified to regulate HEV infection. To further facilitate our understanding on HEV-host interactions, we utilized two HEV cell culture models, combined with proteomics approaches (SILAC and AP-MS) to uncover viral ORF1 associated host factors that regulate viral replication. A variety of host factors were identified from these screens, such as KIF11, ANKFY1, STK38, HSD17B10, KCTD5, PRMT5 and WDR77. As PRMT5 complexed with WDR77 as arginine methyltransferase [48], we focused on the role of this complex in HEV replication. We found that PRMT5/WDR77 complex could interact with HEV ORF1 protein and catalyze methylation at residue R458 of ORF1, which lead to restriction on HEV replication.

Protein arginine methyltransferases (PRMTs), a family of enzymes that catalyze the transfer of a methyl group from S-adenosylmethionine (SAM) to a variety of protein substrates, contribute to regulating a series of cellular biological processes, including signal transduction, RNA processing and transcription [36]. Host PRMTs could also methylate viral proteins and positively or negatively regulate viral infections. It has been well demonstrated that PRMT1 could inhibit HBV transcription via its methyltransferase activity [53]. Moreover, another study found that PRMT5 promotes cccDNA-bound H4R3me2s activity to inhibit cccDNA transcription in a methyltransferase activity dependent manner, while inhibition of core particle DNA production is methyltransferase-independent by preventing interactions of core protein with pgRNA [31]. In addition, a recent study demonstrated that PRMT1 methylates SARS-CoV-2 nucleocapsid (N) protein, and this methylation of N protein is required for its RNA binding capacity to package viral genomic RNA into virion [54]. It has been reported that PRMT5 and WDR77 are able to form the methyltransferase complex in the cytoplasm, catalyzing methylations on proteins and thereby regulating their functions [55–57]. Herein, our study discovered the novel function of PRMT5/WDR77 complex that is able to methylate HEV ORF1 replicase, and thereby restricts HEV replication.

Viruses have always evolved mechanisms against the antiviral effect of host restriction factors. Given that arginine methylation is an important mechanism for regulation of viral protein function, it is therefore not surprising that viral proteins are able to inhibit arginine methyltransferase activity. For example, HBV X protein could counteract the repressive effect of PRMT1 on HBV transcription by inhibiting its catalytic activity [53]. In this study, PRMT5/WDR77 could specifically restrict HEV replication, and it is conceivable that HEV possessed mechanisms to relieve its restriction accordingly, which is worthy of future investigations.

It is important to point out that our study has limitations. Firstly, the PRMT5/WDR77 mediated antiviral effect is not very potent. As shown in Fig 5, depletion of endogenous PRMT5/WDR77 results in 2–3 folds increase of HEV replication *in vitro*. It will be worthy to demonstrate their antiviral activity *in vivo* in the future. Secondly, the interaction of PRMT5/WDR77 with viral ORF1 proteins was poorly characterized due to the technical difficulties that we could not purify the viral ORF1 proteins. Future studies will be needed to reconstitute the methylation of PRMT5/WDR77 on viral ORF1 proteins *in vitro*. As knockdown of PRMT5 leading to instability of WDR77 and *vice versa* (**Fig 5A**), we could not exactly validate the precise interaction mechanism of ORF1 with PRMT5/WDR77 complex. However, WDR77 is responsible for recruitment of the substrates to the complex for methylation by PRMT5 [24]. It is conceivable that ORF1 interacts with WDR77 (**Fig 2F**), and subsequently methylated by PRMT5/WDR77 complex. Thirdly, the molecular mechanism of restriction of PRMT5/WDR77 on HEV replication is not fully understood. Future studies will be performed to investigate whether the ORF1 methylation affects its replicase activity or its interaction with RNA substrates or other critical steps in viral replication step.

In summary, we combined proteomics approaches with functional testing to identify PRMT5/WDR77 methyltransferase complex as a host restriction factor to inhibit HEV infection. Our study not only promotes more comprehensive understanding of viral infections but also provides therapeutic targets for intervention.

## Materials and methods

### Cell cultures

HEK293T (CRL-3216, ATCC), HepG2C3A (CRL-10741, ATCC), Caco-2 (HTB-37, ATCC) and S10-3 cells (kindly gift from Dr. Suzanne U. Emerson of the National Institutes of Health) were maintained in Dulbecco’s modified Eagle medium (DMEM) (C11965500CP, Gibco) supplemented with 10% (vol/vol) fetal bovine serum (FBS) (SE100-011, VISTECH), and 50 IU/ml penicillin/streptomycin (30-002-CI, Corning) in a humidified 5% (vol/vol) CO_2_ incubator at 37°C. Cells were tested routinely and found to be free of mycoplasma contamination.

### Plasmid construction

plentiCRISPRv2 vector (78852, Addgene) was digested by BsaI restriction enzyme (R3733, NEB) to prepare backbone, then annealed sgRNA oligo was ligated with backbone by T4 DNA ligase (M0202, NEB) to construct knockout sgRNA. sgRNA target sequences are listed in S3 Table. The cDNAs of GFP, ORF1, PRMT5 and WDR77 were constructed into pLVX-IRES-zsGreen vector with 2×MultiF Seamless Assembly Mix (RK21020, ABclonal).

### Lentivirus production and transduction

Vesicular stomatitis virus G protein (VSV-G) pseudotyped lentiviruses were produced by transient transfection of the third-generation packaging plasmids pMD2G (12259, Addgene) and psPAX2 (12260, Addgene) and the transfer vector with VigoFect DNA transfection reagent (T001, Vigorous) into HEK293T cells. The supernatant was changed 12 h post transfection. Supernatants were collected at 36, 60 and 84 h after transfection, pooled, passed through a 0.45 μm filter, aliquoted, and frozen at -80°C. Cells are transduced with lentivirus in the presence of 10 μg/ml polybrene.

### SILAC and mass spectrometry

HepG2C3A cells expressing GFP-Flag or ORF1-Flag were cultured in light (^12^C, ^14^N) or heavy (^13^C, ^15^N) SILAC medium separately for two weeks to thoroughly label lysine and arginine. Labelled amino acids were purchased from Cambridge Isotope Laboratories, Inc. Then, 3×10^7^ SILAC labelled cells were collected, washed with DPBS once and lysed by cell lysis buffer (50 mM Tris-HCl [pH 7.5], 150 mM NaCl, 1 mM EDTA, 1% NP-40) supplied with 1 mM dithiothreitol (DTT) (0281, AMRESCO), 1 mM PMSP (36978, Thermo Fisher Scientific) and 1:100 protease inhibitor (P8340, Sigma). Cells were lysed for 30 minutes at 4°C with constant rotation, and the lysates were centrifugated at 17000×g and 4°C for 15 minutes. 50 μl whole-well lysate was collected for western blot analysis. The remaining supernatants were incubated with magnetic beads bound to anti-Flag M2 monoclonal antibody (M8823, Sigma) at 4°C overnight. Beads were washed with cell lysis buffer for 5 times, then eluted with 60 μl SDS loading buffer by heating for 10 minutes at 95°C. 20 μL eluate was removed for Coomassie blue staining to verify whether immunoprecipitation has worked. After verifying protein purification, 20 μL eluate of GFP-Flag light or heavy is mixed with ORF-Flag heavy or light. Mixed protein eluate was stained by Coomassie blue, cut and digested with trypsin to a Orbitrap Fusion mass spectrometer (Thermo Fisher Scientific). Two independent biological replicates are performed for each Flag-tagged protein.

### Replication complex purification

HEK293T cells transfected with Kernow C1/p6 ORF1HA-Flag BSR2AGluc RNA were selected with 5 μg/ml blasticidin (R21001, Thermo Fisher Scientific) for 7 days. 1×10^8^ HEK293T Kernow C1/p6 ORF1HA-Flag BSR2AGluc replicon cells were collected by trypsin digestion and washed with DPBS. Cells were pelleted and lysed with cell lysis buffer (50 mM Tris-HCl [pH 7.5], 150 mM NaCl, 1 mM EDTA, 1% NP-40) supplied with 1 mM DTT, 1 mM PMSP and 1:100 protease inhibitor. Cell lysates were centrifuged at 17000×g for 15 minutes at 4°C. The supernatant was removed and incubated with 150 μl magnetic beads bound to anti-Flag M2 monoclonal antibody (M8823, Sigma) overnight at 4°C. Beads were washed with wash buffer (50 mM Tris-HCl [pH 7.5], 150 mM NaCl, 1 mM EDTA, 1% NP-40) for 5 times and eluted with 60 μl 150 μg/mL 3×Flag peptides (diluted in wash buffer) for 15 min on ice.

### In vitro transcription assay and viral RNA transfection

HEV KernowC1/p6, HEV KernowC1/p6-Gluc, and the mutant replicon plasmids were linearized by MluI. pSAR55-Gluc was linearized by BglII, pGEM-9Zf-pSHEV3-Gluc was linearized by XbaI. Linearized plasmid templates were transcribed *in vitro* with HiScribe T7 antireverse cap analog (ARCA) mRNA kit (E2065S, NEB) according to the manufacturer’s instructions. Viral RNA was transfected into HepG2C3A cells or S10-3 cells using TransIT-mRNA transfection reagent (MIR 2225, Mirus Bio) according to the instructions.

### Gaussia luciferase assays

Gaussia luciferase activity was measured using Renilla Luciferase Assay System (E2820, Promega). Collected supernatant was lysed with 5×lysis buffer for 15 minutes. Then, 10 μl sample was added into 96-well polystyrene microplate (Corning), followed by the addition of Renilla luciferase assay working solution and the detection of luminescence was performed on a GloMax Discover System (Promega).

### HEV production, infection and titration

S10-3 cells were seeded into 6-well plate one day before transfection. Cells were transfected with Kernow C1/p6 RNAs and incubated at 37°C for 7 days. For virus collection, cells were trypsinized and pelleted in a 1.5-ml tube by centrifugation, liquid was removed. Cells were lysed by adding 0.6 ml sterilized H_2_O and put on ice for 30 min, further lysed by freeze-thawing (-80°C-25°C) for 3 times. Cellular debris was removed by centrifugation at 17000×g for 15 min, and the supernatant was supplemented with 1/10 volume of 10×PBS. For virus infection, HepG2C3A cells were seeded into 24-well plate one day before infection at 30% confluence. Cells were infected with virus in 4% PEG8000 (P8260, Solarbio) culture medium. The virus mixture was removed one day later, and cell culture medium containing 2% DMSO (P8340, Sigma) was added, followed by incubation at 37°C for 5 days. For viral titration, HepG2C3A cells seeded in 96-well plate (10,000 cells per well) one night before were infected with diluted HEV. The inoculum was removed 12 hours post infection and cells were cultured in fresh culture medium supplied with 2% DMSO. Three days post infection, cells were fixed and stained with ORF2 antibody (2G8) [20]. The number of ORF2 positive cells was counted and used to calculate infectious titers.

### Immunofluorescence

Cells in 24-well plate were fixed with 4% paraformaldehyde at room temperature (RT) for 20 min. The cells were permeabilized in 0.25% triton X-100 (H5142, Promega) for 20 minutes and blocked in 1% BSA (180728, MP) for 1 hour at RT. Cells were stained by ORF2 antibody (2G8) [20,58], HA (M20003, Abmart) in 1% BSA and incubated at RT for 1 hour, then cells were washed with PBS three times. Goat anti-mouse IgG (H+L) secondary antibody Alexa Fluor 488 conjugate (A11029, Invitrogen) diluted in 1% BSA was used to stain cells at RT for another 1 hour, then cells were washed with PBS three times. Nucleus was stained by 1 μg/ml DAPI (32670, Sigma) for 5 minutes at RT, then cells were washed with PBS three times. The stained cells were observed under microscope (Nikon Eclipse Ti2).

### Flow cytometry analysis

Cells were digested with 0.25% trypsin and stopped by cell culture medium. Cells were centrifuged at 500×g for 5 minutes at 4°C, then washed with DPBS once. Cells were fixed in 4% Paraformaldehyde (PFA) for 30 minutes at room temperature. Cells were permeabilized with 0.25% triton X-100 for 30 minutes and blocked in 1% BSA for 1 hour at room temperature. Cells were incubated with primary antibody (anti-ORF2 (2G8) or anti-WDR77 (2018S, CST) for 1.5 hours at room temperature. After washed with PBS for three times, cells were incubated with Goat anti-mouse/rabbit IgG (H+L) secondary antibody (A11029/A11036, Invitrogen) for 1 hour. Cells were washed with PBS three times and resuspended in PBS. Data were collected with LSRFortessa SORP (BD Biosciences) and analyzed by FlowJo software (BD Biosciences).

### Sample preparation and methylation analysis of ORF1 by mass spectrometry

pLVX-ORF1-Flag-IRES-zsGreen plasmid was transfected into HEK293T cells. Cells were collected at 72 hours post transfection and lysed with cell lysis buffer (50 mM Tris-HCl [pH 7.5], 150 mM NaCl, 1 mM EDTA, 1% NP-40). ORF1-Flag was immunoprecipitated by Flag antibody and washed with cell lysis buffer. ORF1 was eluted by heating the beads at 95°C for 5 minutes. Gel bands of proteins were excised for in-gel digestion, and proteins were identified by mass spectrometry. Briefly, proteins were disulfide reduced with 25 mM DTT and alkylated with 55 mM iodoacetamide (1632109, Bio-Rad). In-gel digestion was performed using sequencing grade-modified trypsin in 50 mM ammonium bicarbonate (A6141, Sigma) at 37°C overnight. The peptides were extracted twice with 1% trifluoroacetic acid (400445, Thermo Fisher Scientific) in 50% acetonitrile aqueous solution for 30 min. The peptide extracts were then centrifuged in a SpeedVac (Thermo Fisher Scientific) to reduce the volume. For LC-MS/MS analysis, peptides were separated by a 60 min gradient elution at a flow rate 0.3 μl/min with a Thermo-Dionex Ultimate 3000 HPLC system, which was directly interfaced with the Thermo Orbitrap Fusion mass spectrometer. The analytical column was a homemade fused silica capillary column (75 μm ID, 150 mm length; Upchurch, Oak Harbor, WA) packed with C-18 resin (300 A, 5 μm; Varian, Lexington, MA). Mobile phase A consisted of 0.1% formic acid, and mobile phase B consisted of 100% acetonitrile and 0.1% formic acid. The Orbitrap Fusion mass spectrometer was operated in the data-dependent acquisition mode using Xcalibur3.0 software (Thermo Fisher Scientific) and there is a single full-scan mass spectrum in the Orbitrap (350–1550 m/z, 120,000 resolution) followed by 3 seconds data-dependent MS/MS scans in an Ion Routing Multipole at 30% normalized collision energy (HCD). The MS/MS spectra from each LC-MS/MS run were searched against the selected database using Proteome Discovery searching algorithm (version 1.4) [59]. A precursor mass tolerance of 20 ppm and a product ion tolerance of 0.02 Da were allowed. One tryptic terminus was required, and two missed cleavages were allowed. Static carbamidomethylation of cysteine (+57.02146374) was required, and appropriate arginine modifications (+14.0156500642 for monomethyl and +28.0313001284 for dimethyl) and methionine oxidation (+15.9949146221) were dynamically allowed with a maximum of four modifications of one type per peptide. Peptide spectral matches were filtered to a 1% false discovery rate using linear discriminant analysis in combination with the target-decoy strategy.

### Western blotting assay

Sodium dodecyl sulfate-polyacrylamide gel electrophoresis (SDS-PAGE) immunoblotting was performed as follows: After trypsinization and cell pelleting at 500×g for 5 min, whole-cell lysates were harvested in cell lysis buffer (50 mM Tris-HCl [pH 7.5], 150 mM NaCl, 1 mM EDTA, 1% NP-40) supplemented with protease inhibitor (P8340, Sigma). Lysates were electrophoresed in 4–12% polyacrylamide gels and transferred onto PVDF membrane (IPVH00010, Millipore). The blots were blocked at room temperature for 0.5 h using 5% non-fat milk in PBS containing 0.1% (v/v) Tween 20. The blots were exposed to primary antibodies, β-Tubulin (CW0098, CWBIO), β-actin (AM1021b, Abcepta), Flag (F7425, Sigma), PRMT5 (sc-376937, Santa Cruz), WDR77 (2018S, CST), HA-HRP (M20021, Abmart) in 5% nonfat milk in 1×PBS containing 0.1% Tween 20 for 1.5 h. The blots were then washed in 1×PBS containing 0.1% Tween 20. After 1h exposure to HRP-conjugated secondary antibodies (AS003 or AS014, ABclonal) and subsequent washes were performed as described for the primary antibodies. Membranes were subsequently development with enhanced chemiluminescence (ECL) substrate (BE6706, Easybio) and visualized using the Luminescent Image Analyzer (GE).

### RT-qPCR

Cells were washed with DPBS once and lysed with 500 μl trizol (15596018, Invitrogen) for 5 minutes at RT. Then, 100 μl chloroform was added and mixed thoroughly. Samples were centrifuged at 17000×g and 4°C for 15 minutes. The supernatant was removed into a new tube and added with equal volume of isopropyl alcohol. Samples were centrifuged at 17000×g and 4°C for another 15 minutes. RNA pellets were washed with 75% ethanol twice and eluted with 50 μl nuclease free water. RNA concentration was measured by spectrophotometer (Nanodrop, Thermo Fisher Scientific). For reverse transcription, 1 μg total RNA was reverse transcribed using ReverTra Ace qPCR RT Kit (FSQ-101, TOYOBO). Reactions were carried out using the 2×RealStar Green Power Mixture (A311, Genstar) according to the instruction. Relative expression levels of the target genes were calculated using the comparative cycle threshold (CT) method. Host and HEV are normalized relative to the housekeeping gene GAPDH (glyceraldehyde-3-phosphate dehydrogenase). RT-PCR primers of target genes were listed in S3 Table.

### Statistical analysis

Student’s *t* test or one-way analysis of variance (ANOVA) with Tukey’s honestly significant difference (HSD) test was used to test for statistical significance of the differences between the different group parameters. *P* values of less than 0.05 were considered statistically significant.

## Supporting information

S1 Fig Identification of host proteins interacting with ORF1 in ORF1 *trans*-complementation and replicon systems. (A) Western blot analysis of lysates from HepG2C3A cells transduced with GFP-Flag or ORF1-Flag lentiviruses. Tubulin was used as the loading control. (B) Kernow C1/p6 Gluc GAD (RdRp inactive) replicon RNAs were transfected into HepG2C3A expressing GFP-Flag or ORF1-Flag cells, and Gluc activity was measured at day 2 post transfection. (C) Coomassie blue staining of whole-cell lysate and immunoprecipitated samples from GFP-Flag and ORF1-Flag expressing HepG2C3A cells, red star indicating proteins interacting with ORF1 specifically. L, light; H, heavy. (D) Kernow C1/p6 Gluc WT or HA-Flag insertion replicon RNAs were transfected into S10-3 cells. Supernatants from each group were collected and Gluc activity was measured at day 2 post transfection. GAD is RdRp inactive control. (E) Western blot analysis of lysates from HEK293T cells transfected with Kernow C1/p6 ORF1HA-Flag BSR2AGluc replicon. Tubulin was used as the loading control. (F) Coomassie blue staining of immunoprecipitated HEV replication complex from Kernow C1/p6 ORF1HA-Flag BSR2AGluc replicon cells. Immunoprecipitation using cells expressing SARS-CoV-2 N-Flag was used as the control. Lane 1–4 indicated different elution in the purification process. Specific interacted proteins are listed on the left. Values are means plus standard deviations (SD) (error bars) (n = 3). ***, P < 0.001; n.s., not significantly different by Student’s t test. All data are representative of three independent experiments.
(TIF)

S2 Fig HEV infection does not alter WDR77 expression and subcellular localization. (A) HEK293T cells were transfected with HA-PRMT5 cDNA. Cells were stained with WDR77 and HA antibodies prior to analysis by confocal microscopy. The cell nuclei were stained with DAPI (blue). Line profiles corresponding to the white lines show colocalization. (B) HepG2C3A cells were infected with Kernow C1/p6 and RT-qPCR analysis of PRMT5 and WDR77 mRNA was performed at day 3 post infection. Data are normalized with mock control. (C) HepG2C3A cells were infected with Kernow C1/p6 and flow cytometry analysis of WDR77 was performed at day 3 post infection. (D) HepG2C3A cells were infected with Kernow C1/p6 and immunofluorescence of ORF2, WDR77 was performed at day 3 post infection. Values are means plus standard deviations (SD) (error bars) (n = 3). n.s., not significantly different by Student’s t test. All data are representative of three independent experiments.
(TIF)

S3 Fig PRMT5/WDR77 complex inhibits HEV infection. Wild-type or PRMT5 knockdown S10-3 cells were infected with Kernow C1/p6. Flow cytometry analysis of HEV infection was performed at day 3 post infection. Data are representative of three independent experiments.
(TIF)

S4 Fig PRMT5/WDR77 complex restricts HEV replication. WT or PRMT5, WDR77 knockdown S10-3 cells were transfected with HEV replicon RNA. HEV genomic RNAs were analyzed at day 4 post transfection of Kernow C1/p6 Gluc replicon (A), Sar55 Gluc replicon (B) and pSHEV3 Gluc replicon (C). Data are normalized with non-targeting control sgRNA. Values are means plus standard deviations (SD) (error bars) (n = 3). *, P < 0.05; **, P < 0.01; ***, P < 0.001 by one-way ANOVA.
(TIF)

S5 Fig Mass spectrometry analysis identifies methylation of R80, R255, R458, R560, R564, R1079 in ORF1. pLVX-ORF1-Flag-IRES-zsGreen plasmid was transfected into HEK293T cells for 72 h. The transfected cells were subjected to IP using Flag antibody to purify ORF1-Flag. Purified ORF1-Flag was separated by SDS-PAGE and visualized by CBB staining. The specific ORF1-Flag band was excised, digested, and analyzed by liquid chromatography-mass spectrometry (LC-MS). Representative tandem mass spectrum of peptides showing that R80, R255, R458, R560, R564 and R1079 were methylated.
(TIF)

S6 Fig PRMT5/WDR77 complex catalyzes ORF1 R458 methylation to inhibit HEV replication. (A) WT, PRMT5 knockdown or WDR77 knockdown S10-3 cells were transfected with Kernow C1/p6 Gluc WT or R458K mutant replicon RNAs. Gluc activity was measured at day 2 post transfection. Data were normalized with S10-3 WT cells transfected with Kernow C1/p6 Gluc WT replicon RNAs. (B) S10-3 cells overexpressing PRMT5 and WDR77 were transfected with Kernow C1/p6 RNA and intracellular HEV virions were titrated on day 7 post transfection in HepG2C3A cells. Values are means plus standard deviations (SD) (error bars) (n = 3). *, P < 0.05 by Student’s t test. (C) Conservation analysis of 458^th^ methylated arginine of Kernow C1/p6 ORF1 among eight HEV genotypes in the species of *Paslahepevirus balayani*.
(TIF)

S1 Table ORF1 interactome identified by SILAC-MS, ORF1/GFP >3 and score>5 host factors. (XLSX)

S2 Table Host proteins in ORF1 replication complex identified by AP-MS. (XLSX)

S3 Table RT-qPCR primers and sgRNA target sequences. (XLSX)
