# Supplementary figures and images for: The PRMT5/WDR77 complex restricts hepatitis E virus replication
Source: PLoS Pathog. 2023 Jun 5;19(6):e1011434. doi: 10.1371/journal.ppat.1011434 (PMC10270597; doi:10.1371/journal.ppat.1011434)

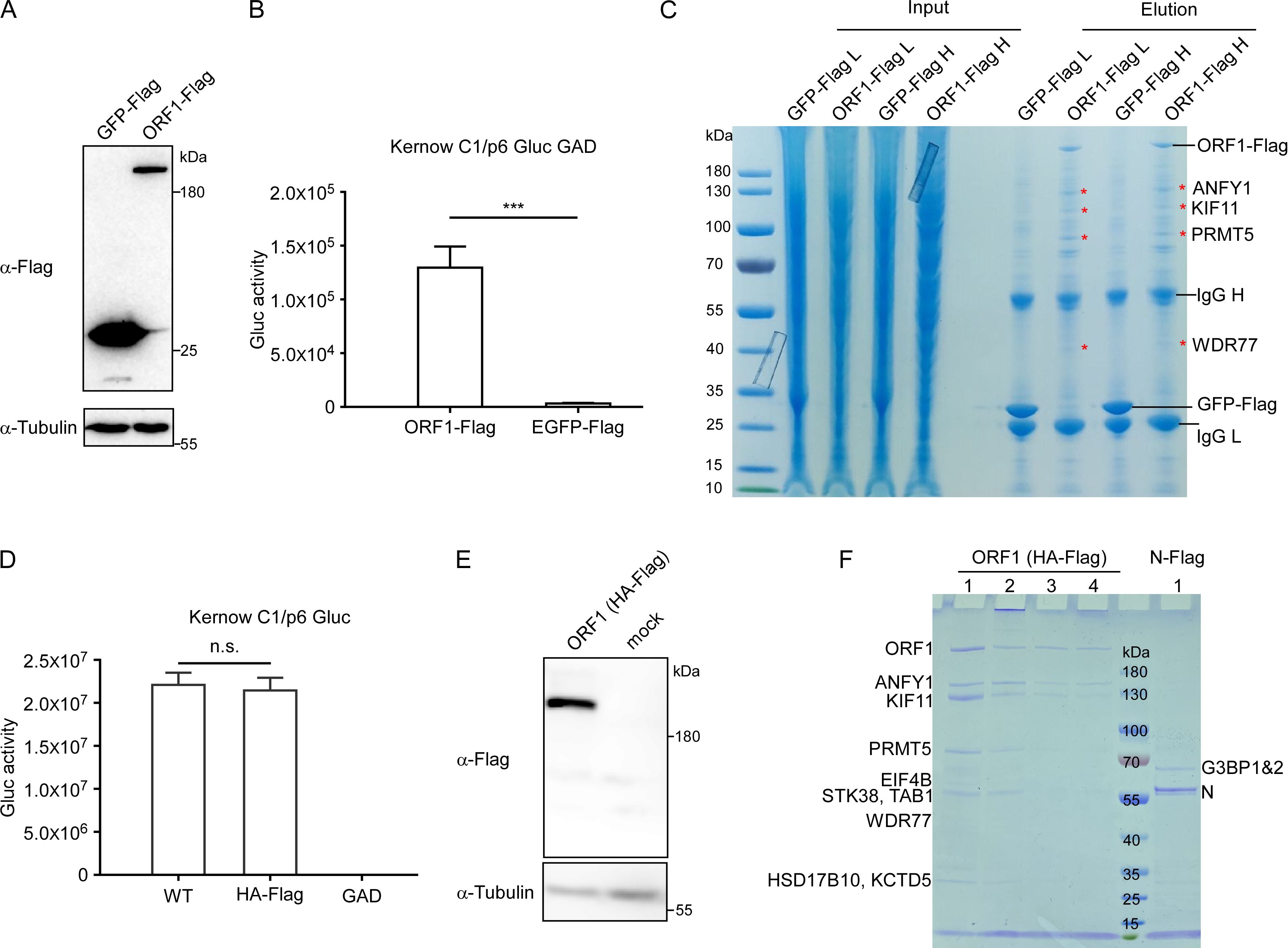

Supplement: S1 Fig — (A) Western blot analysis of lysates from HepG2C3A cells transduced with GFP-Flag or ORF1-Flag lentiviruses. Tubulin was used as the loading control. (B) Kernow C1/p6 Gluc GAD (RdRp inactive) replicon RNAs were transfected into HepG2C3A expressing GFP-Flag or ORF1-Flag cells, and Gluc activity was measured at day 2 post transfection. (C) Coomassie blue staining of whole-cell lysate and immunoprecipitated samples from GFP-Flag and ORF1-Flag expressing HepG2C3A cells, red star indicating proteins interacting with ORF1 specifically. L, light; H, heavy. (D) Kernow C1/p6 Gluc WT or HA-Flag insertion replicon RNAs were transfected into S10-3 cells. Supernatants from each group were collected and Gluc activity was measured at day 2 post transfection. GAD is RdRp inactive control. (E) Western blot analysis of lysates from HEK293T cells transfected with Kernow C1/p6 ORF1HA-Flag BSR2AGluc replicon. Tubulin was used as the loading control. (F) Coomassie blue staining of immunoprecipitated HEV replication complex from Kernow C1/p6 ORF1HA-Flag BSR2AGluc replicon cells. Immunoprecipitation using cells expressing SARS-CoV-2 N-Flag was used as the control. Lane 1–4 indicated different elution in the purification process. Specific interacted proteins are listed on the left. Values are means plus standard deviations (SD) (error bars) (n = 3). ***, P < 0.001; n.s., not significantly different by Student’s t test. All data are representative of three independent experiments. (TIF) [file ppat.1011434.s001.tif]

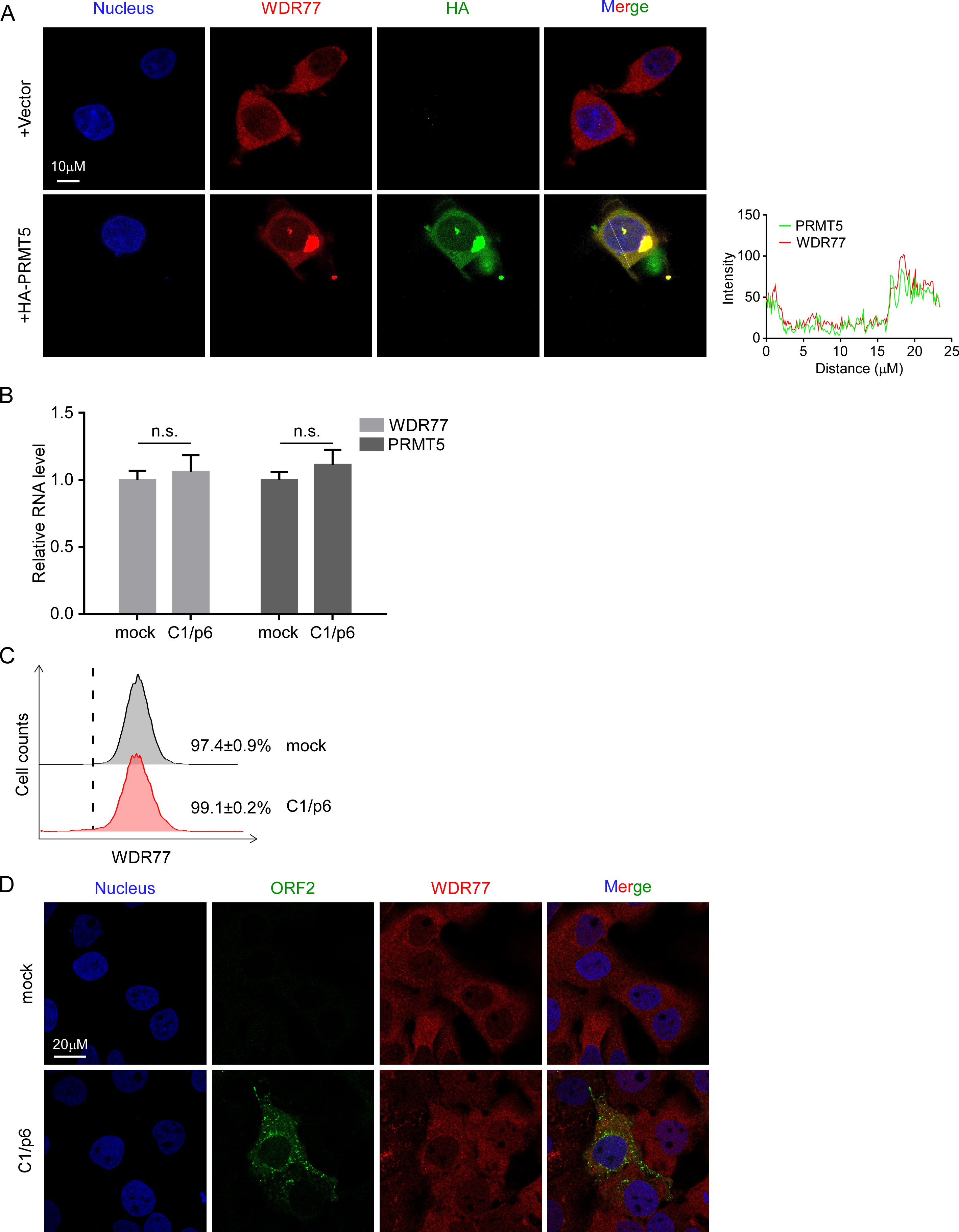

Supplement: S2 Fig — (A) HEK293T cells were transfected with HA-PRMT5 cDNA. Cells were stained with WDR77 and HA antibodies prior to analysis by confocal microscopy. The cell nuclei were stained with DAPI (blue). Line profiles corresponding to the white lines show colocalization. (B) HepG2C3A cells were infected with Kernow C1/p6 and RT-qPCR analysis of PRMT5 and WDR77 mRNA was performed at day 3 post infection. Data are normalized with mock control. (C) HepG2C3A cells were infected with Kernow C1/p6 and flow cytometry analysis of WDR77 was performed at day 3 post infection. (D) HepG2C3A cells were infected with Kernow C1/p6 and immunofluorescence of ORF2, WDR77 was performed at day 3 post infection. Values are means plus standard deviations (SD) (error bars) (n = 3). n.s., not significantly different by Student’s t test. All data are representative of three independent experiments. (TIF) [file ppat.1011434.s002.tif]

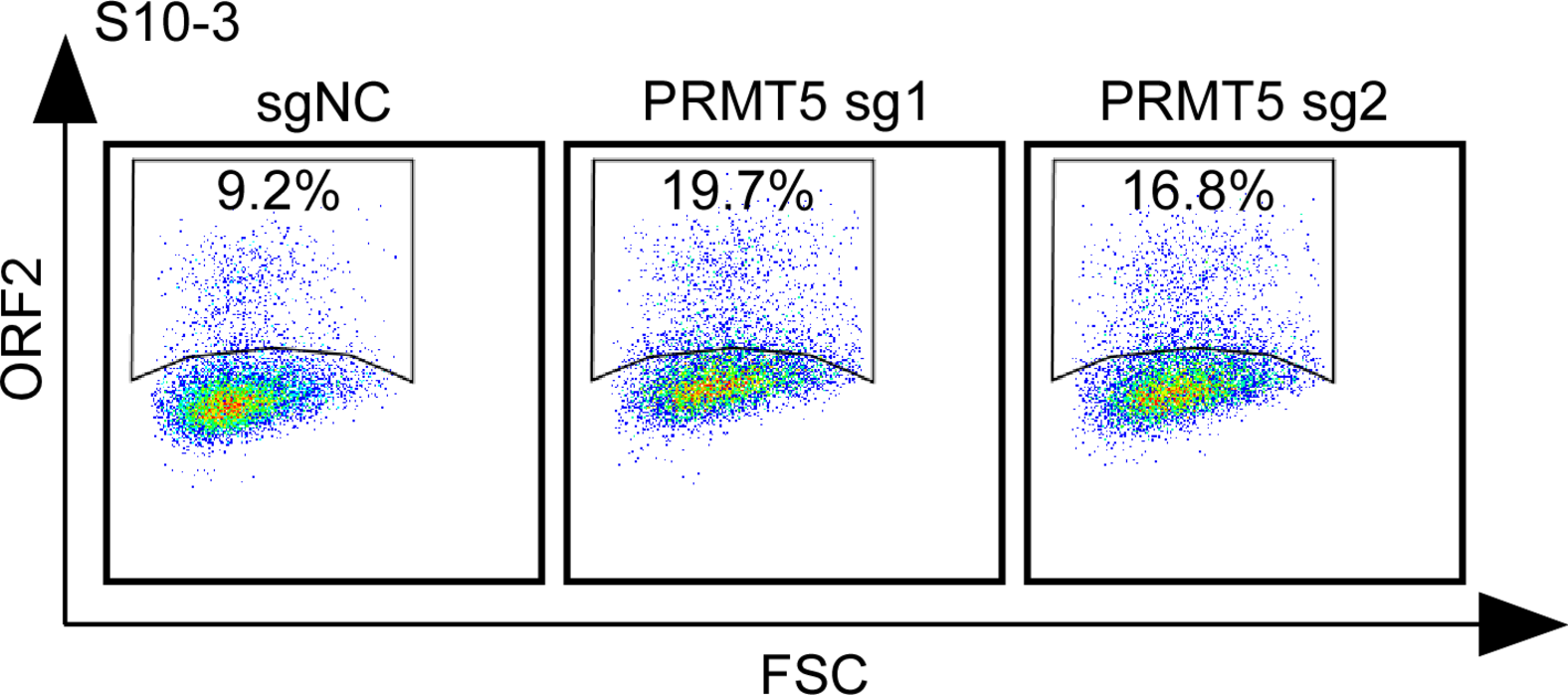

Supplement: S3 Fig — Wild-type or PRMT5 knockdown S10-3 cells were infected with Kernow C1/p6. Flow cytometry analysis of HEV infection was performed at day 3 post infection. Data are representative of three independent experiments. (TIF) [file ppat.1011434.s003.tif]

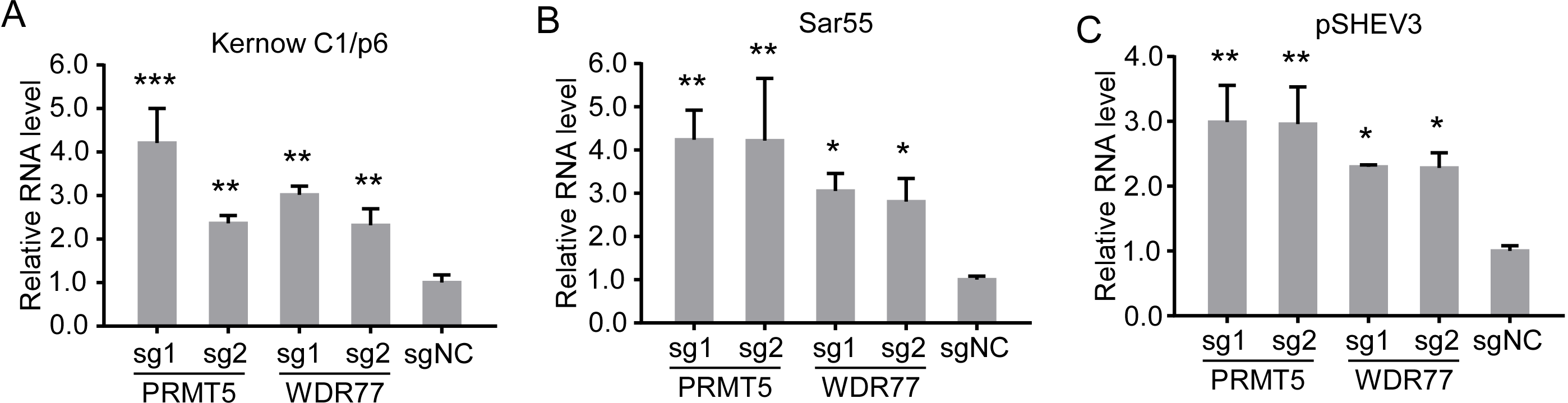

Supplement: S4 Fig — WT or PRMT5, WDR77 knockdown S10-3 cells were transfected with HEV replicon RNA. HEV genomic RNAs were analyzed at day 4 post transfection of Kernow C1/p6 Gluc replicon (A), Sar55 Gluc replicon (B) and pSHEV3 Gluc replicon (C). Data are normalized with non-targeting control sgRNA. Values are means plus standard deviations (SD) (error bars) (n = 3). *, P < 0.05; **, P < 0.01; ***, P < 0.001 by one-way ANOVA. (TIF) [file ppat.1011434.s004.tif]

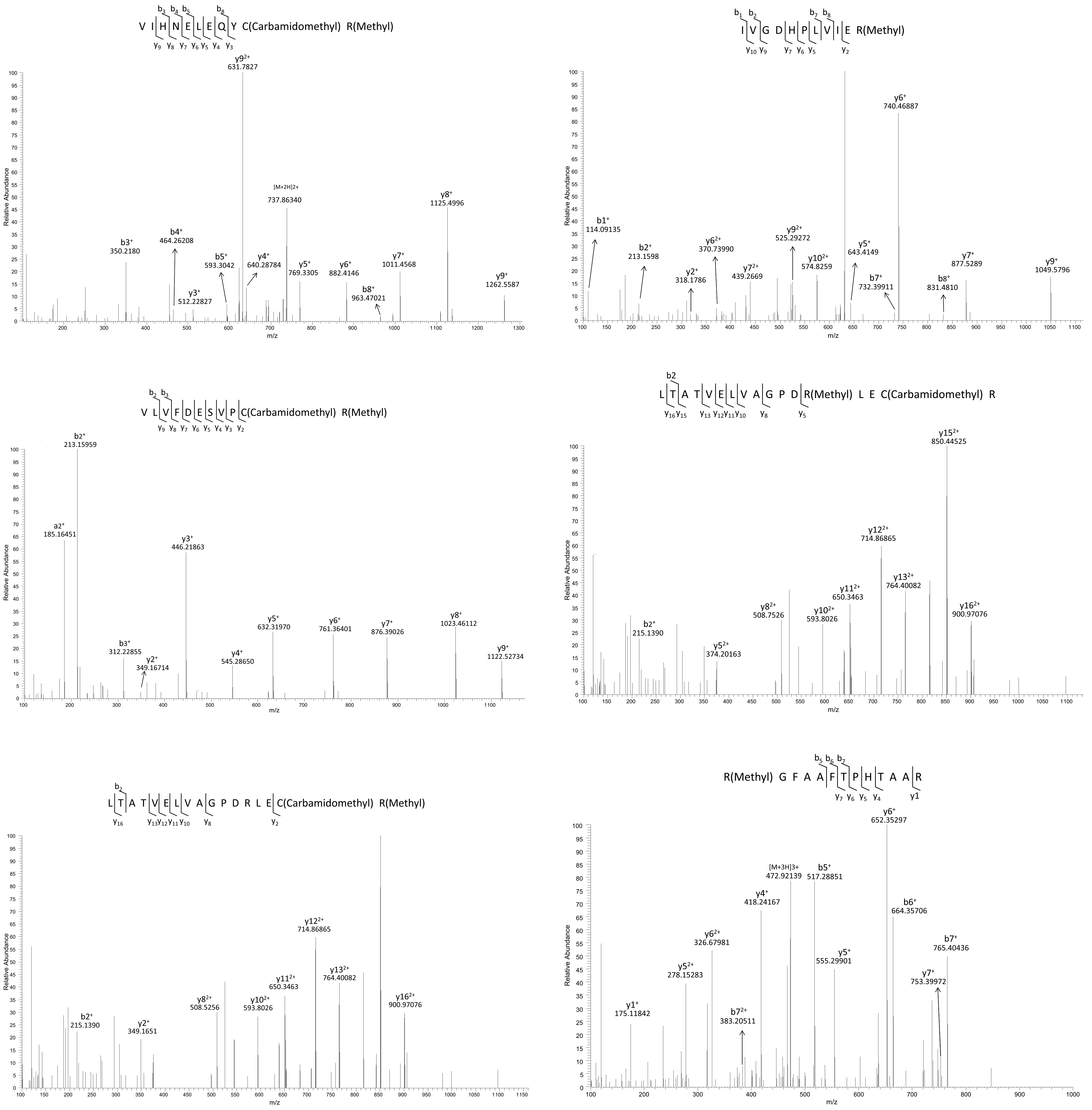

Supplement: S5 Fig — pLVX-ORF1-Flag-IRES-zsGreen plasmid was transfected into HEK293T cells for 72 h. The transfected cells were subjected to IP using Flag antibody to purify ORF1-Flag. Purified ORF1-Flag was separated by SDS-PAGE and visualized by CBB staining. The specific ORF1-Flag band was excised, digested, and analyzed by liquid chromatography-mass spectrometry (LC-MS). Representative tandem mass spectrum of peptides showing that R80, R255, R458, R560, R564 and R1079 were methylated. (TIF) [file ppat.1011434.s005.tif]

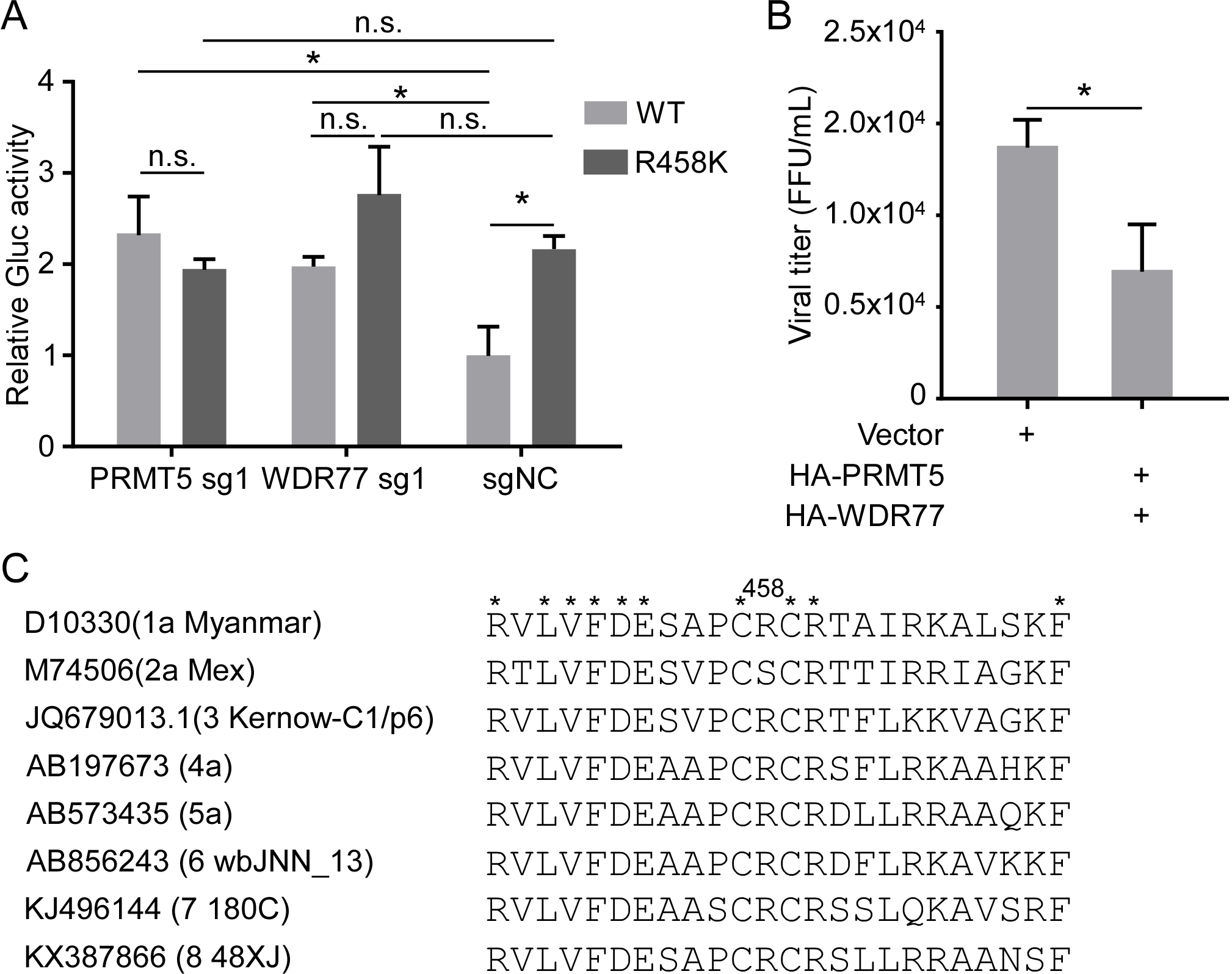

Supplement: S6 Fig — (A) WT, PRMT5 knockdown or WDR77 knockdown S10-3 cells were transfected with Kernow C1/p6 Gluc WT or R458K mutant replicon RNAs. Gluc activity was measured at day 2 post transfection. Data were normalized with S10-3 WT cells transfected with Kernow C1/p6 Gluc WT replicon RNAs. (B) S10-3 cells overexpressing PRMT5 and WDR77 were transfected with Kernow C1/p6 RNA and intracellular HEV virions were titrated on day 7 post transfection in HepG2C3A cells. Values are means plus standard deviations (SD) (error bars) (n = 3). *, P < 0.05 by Student’s t test. (C) Conservation analysis of 458th methylated arginine of Kernow C1/p6 ORF1 among eight HEV genotypes in the species of Paslahepevirus balayani. (TIF) [file ppat.1011434.s006.tif]
